# Noninvasive and Reliable Quantification of Anteromedial Rotatory Knee Laxity: A Pilot Study on Healthy Individuals

**DOI:** 10.1177/03635465241234263

**Published:** 2024-03-20

**Authors:** Lukas Willinger, Armin Runer, Romed Vieider, Lukas N. Muench, Sebastian Siebenlist, Philipp W. Winkler

**Affiliations:** * Department of Sports Orthopaedics, Technical University of Munich, Munich, Germany; † Department of Trauma and Reconstructive Surgery, Artemed Klinikum München Süd, Munich, Germany; § Department of Orthopaedics and Traumatology, Kepler University Hospital Linz, Linz, Austria; Investigation performed at the Department of Sports Orthopaedics, Technical University of Munich, Munich, Germany

**Keywords:** knee, MCL, laxity, digital, anteromedial, instability

## Abstract

**Background:**

Anteromedial rotatory instability (AMRI) of the knee is a complex and severe condition caused by injury to the anterior cruciate ligament and/or the medial collateral ligament. Clinical studies dealing with AMRI are rare, and objective measurements are nonexistent.

**Purpose/Hypothesis:**

The objectives of this study were, first, to quantify anteromedial rotatory knee laxity in healthy individuals using a noninvasive image analysis software and, second, to assess intra- and interrater reliability and equivalence in measuring anteromedial knee translation (AMT). It was hypothesized that AMT could be reliably quantified using a noninvasive image analysis software.

**Study design:**

Cohort study; Level of evidence, 3.

**Methods:**

This prospective proof-of-concept study included healthy individuals aged 16 to 40 years with no history of knee injury or surgery. Three adhesive surface markers were placed on predefined landmarks on the medial side of the knee. Three independent investigators examined anteromedial rotatory knee laxity with an anterior drawer test in different tibial rotations (neutral tibial rotation, 15° of external tibial rotation, and 15° of internal tibial rotation). The entire examination of each knee was recorded, and AMT including the side-to-side difference (SSD) was assessed using a freely available and validated image analysis software (PIVOT iPad application). Group comparisons were performed using a 1-way analysis of variance with Bonferroni-adjusted post hoc analysis. Intraclass correlation coefficients (ICCs) were calculated to assess inter- and intrarater reliability of AMT measurements. Equivalence of measurements was evaluated using the 2 one-sided *t*-test procedure.

**Results:**

Anteromedial rotatory knee laxity was assessed in 30 knees of 15 participants (53% male) with a mean age of 26.2 ± 3.5 years. In all 3 raters, the highest AMT was observed in neutral tibial rotation (range of means, 2.2-3.0 mm), followed by external tibial rotation (range of means, 2.0-2.4 mm) and internal tibial rotation (range of means, 1.8-2.2 mm; *P* < .05). Intrarater reliability of AMT (ICC, 0.88-0.96) and SSD (ICC, 0.61-0.96) measurements was good to excellent and moderate to excellent, respectively. However, interrater reliability was poor to moderate for AMT (ICC, 0.44-0.73) and SSD (ICC, 0.12-0.69) measurements. Statistically significant equivalence of AMT and SSD measurements was observed between and within raters for almost all testing conditions.

**Conclusion:**

Anteromedial rotatory knee laxity could be quantified using a noninvasive image analysis software, with the highest AMT observed during neutral tibial rotation in uninjured individuals. Reliability and equivalence of measurements were good to excellent within raters and moderate between raters.

Anteromedial rotatory instability (AMRI) of the knee is a complex condition characterized by increased rotational movement at the tibiofemoral joint. This condition leads to recurrent episodes of subjective knee instability with the knee giving way and thus causes functional impairment. Acute disruption or chronic insufficiency of the stabilizing structures, in particular the anterior cruciate ligament (ACL) and the superficial and deep medial collateral ligament (MCL), can result in abnormal anterior tibial translation and external tibial rotation.^4,34^

Diagnosing AMRI poses a significant challenge. Physical examination findings, including increased forward displacement of the medial tibial plateau during the Lachman or anterior drawer test with additional external rotation (ie, Slocum test), can aid in the clinical assessment of AMRI.^29,30^ However, these tests depend on the examiner's experience and subjective rating. Objective quantification of knee instability is vital for researchers to compare patients’ preoperative status and evaluate treatment outcomes. New technologies, such as optical or electromagnetic tracking systems, help researchers to assess joint laxity and instabilities.

The PIVOT iPad application (University of Pittsburgh) is a freely available and noninvasive image analysis software that has been shown to accurately and reliably quantify lateral knee compartment translation during the pivot-shift test and scapular motion in patients with scapular dyskinesis.^24,32^ This video-based technology detects relative displacements between surface markers attached to predefined landmarks to determine bony motion. The PIVOT iPad application has successfully assessed anterolateral rotatory knee laxity^24^ and therefore may also be suitable for quantifying anteromedial rotatory knee laxity.

The objectives of this study were to quantify anteromedial rotatory knee laxity in healthy individuals using a noninvasive image analysis software and to assess intra- and interrater reliability and equivalence in measuring anteromedial knee translation (AMT). It was hypothesized that AMT could be reliably quantified using a noninvasive image analysis software.

## Methods

This was a prospective proof-of-concept study approved by the ethics committee of the Technical University of Munich (No. 2022-356-S-SR). Written informed consent was obtained from each participant before enrollment in the study.

Healthy individuals aged 16 to 40 years with no history of knee injury or surgery were consecutively enrolled in this study. Skin disorders in the area of the knee joint, a body mass index (BMI) ≥35, or clinical signs of knee instability, excessive external tibial torsion, or lower limb malalignment led to exclusion from the study. Lower limb alignment was assessed clinically as described previously to avoid unnecessary radiation exposure of healthy individuals.^26^

### Clinical Examination of Anteromedial Rotatory Knee Laxity

Clinical examination of anteromedial rotatory knee laxity was performed by 3 independent investigators: 1 fellowship-trained orthopaedic consultant (L.W.) and 2 fellowship-trained residents (A.R. and P.W.W.). The investigators were blinded to the results of their coinvestigators’ examination. Both knees were examined in each participant.

**Figure 1.**
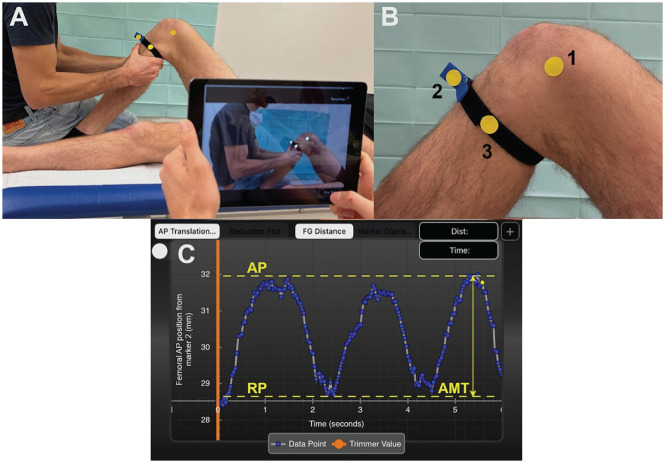
Experimental setup. (A) Recording the anteromedial rotatory knee laxity examination using the PIVOT iPad application. (B) Right knee with attached surface markers on the following landmarks: (1) medial femoral epicondyle, (2) a sagittally aligned clamp that was attached to the most prominent part of the tibial tuberosity, and (3) 8 cm posterior to marker 2. (C) Screenshot of PIVOT iPad application showing quantitative output of 1 test cycle: ie, 3 intervals of anteromedial knee translation (AMT; double arrow) measurement. The *x*-axis represents the time in seconds, and the *y*-axis represents AMT (relative displacement between marker 1 and markers 2 and 3) in mm. Dashed yellow lines represent the maximum anterior position (AP) and the resting position (RP) of the tibia.

Participants were placed supine with the examined knee flexed to 90°. Three yellow adhesive surface markers with a diameter of 19 mm were placed on predefined landmarks on the medial side of the knee. Marker 1 was placed on the medial femoral epicondyle, marker 2 was placed on a sagittally aligned clamp that was attached to the most prominent part of the tibial tuberosity via a strap running around the lower leg, and marker 3 was placed 8 cm posterior to marker 2 (Figure 1).

Examination of anteromedial rotatory knee laxity followed the rotatory instability test described by Slocum and Larson.^30^ During the examination, the investigators sat on the dorsum of the participant's foot to stabilize the leg and thereby maintain the position of the knee. The investigators grasped the tibia with both hands directly distal to the tibial tuberosity to apply an anteriorly directed force to the tibia (ie, anterior drawer test, as used in regular clinical practice). Each examination started with the tibia in a neutral rotation position (with the foot pointed forward), followed by a submaximal external position of 15° of foot rotation and an internal rotation position of 15° of foot rotation. In each rotational position of the tibia, the investigators performed 3 intervals in which the tibia was drawn anteriorly with a submaximal force (comprising 1 test cycle). Utmost care was taken that the hamstring muscles were relaxed during testing. Between the 3 intervals, the tibia was returned to its resting position. The different rotational positions were tested because previous publications suggested that tibial rotation may influence anterior tibial translation due to the differences in tautness of secondary restraints (eg, anterolateral and medial ligament complex).^8,30^

### Quantification of Anteromedial Knee Translation

An independent observer (R.V.) recorded the entire examination of each knee using the PIVOT iPad application Version 1.05. The PIVOT iPad application is a freely available app that can be used on a commercially available iPad (Apple Inc). The application was designed to quantify the translation of the lateral knee compartment during a pivot-shift test using a validated image analysis software.^10,11,23,24^ Strong correlation with 3-dimensional bony motion, good to excellent intrarater agreement, and good interrater agreement in quantifying lateral knee compartment translation underscore the accuracy and reliability of the PIVOT iPad application.^3^ When used according to the developer's recommendations, the mean percentage error in measuring tibial compartment translation is <6%, indicating satisfactory accuracy of the image analysis software.^23^ This study used the PIVOT iPad application to measure the relative motion between the surface marker on the medial femoral epicondyle (marker 1) and the 2 surface markers attached to the tibia (markers 2 and 3). This relative motion indicates the translation of the medial knee compartment and was defined as AMT. To follow the developer's recommendations for the PIVOT iPad application, the iPad was kept at a mean distance of 100 cm (range, 50-175 cm) perpendicular to the surface markers during recording.^23^ Moreover, a monotone background was used to prevent noise interference during video acquisition.^10^

After the video was recorded, the sine curve of 1 test cycle indicating AMT over time could be analyzed on the output screen of the PIVOT iPad application. A sine curve was obtained based on the 3 intervals of anteromedial knee laxity testing. AMT was defined as the distance between the most anterior position and the resting position of the tibia and was determined for each rotational position of the tibia (Figure 1). All data from the PIVOT iPad application were collected by 1 observer (R.V.). Each investigator performed 1 test cycle (consisting of 3 intervals of anteromedial rotatory knee laxity testing) on both knees of each participant. The mean AMT of 1 test cycle was used for data analysis.

### Statistical Analysis

Based on previous studies^8,28^ and a pilot test with 8 healthy knee joints, a difference in AMT of 1 to 2 mm between the different rotational positions of the tibia was considered clinically relevant. The calculation was based on an assumed standard deviation of differences in side-to-side difference (SSD) of 1 mm with equivalence boundaries of ±1 mm (2-sided alpha = .025 and power of 80%). Power analysis was performed with R TOSTER (Version 0.3.4) and two-one-sided *t* test procedure (TOST, Version 1.5-2). The calculation resulted in 11 participants to undergo repeated measurements to achieve adequate power.^28^

Categorical variables were reported as number and percentage. Data distribution of continuous variables was assessed with the Shapiro-Wilk test. Normally distributed data were reported as mean and standard deviation. Nonnormally distributed data were reported as median and interquartile range. Group comparisons (neutral tibial rotation vs external tibial rotation vs internal tibial rotation) were performed with a 1-way repeated-measures analysis of variance with Bonferroni-adjusted post hoc analysis. SSDs were defined as the mean difference in AMT of the right and left knees and were calculated for each rotational position of the tibia.

To evaluate the performance of the PIVOT iPad application in determining AMT, we assessed the equivalence and agreement of measurements between and within the investigators. The equivalence of AMT measurements was assessed using the 2 one-sided *t* test procedure.^18^ The 2 one-sided *t* test procedure is a statistical method to evaluate whether the difference of the obtained measurements falls within predetermined equivalence boundaries and is therefore close to zero, indicating no clinically relevant difference. Based on a previous study that evaluated the equivalence of anterior tibial knee translation measurements with different arthrometers, the upper and lower equivalence boundaries were set at ±1 mm,^28^ which was considered plausible for the present study. Equivalence was assessed by evaluating mean differences of AMT and the corresponding 90% CI between investigators (rater 1 vs rater 2, rater 1 vs rater 3, and rater 2 vs rater 3) and within investigators (AMT of interval 1 vs AMT of interval 2 of 1 test cycle). Measurements obtained by different examiners or at different test times were considered equivalent if the 90% CIs on both sides were found to lie fully within the above-mentioned boundaries of ±1 mm. If confidence intervals were partly inside and partly outside the equivalence range, measurements were considered inconclusive, whereas confidence intervals lying fully outside the boundaries were considered nonequivalent. To evaluate the agreement of measurements, intraclass correlation coefficients (ICCs) were calculated using 2-way mixed-effects models. A small sample size may result in negative ICCs, indicating a low level of agreement, which are accordingly reported as zero.^5,7,19^ Intraclass correlation coefficients <0.5, 0.5 to 0.75, 0.76 to 0.9, and >0.9 were interpreted as poor, moderate, good, and excellent reliability, respectively.^17^

The level of significance was set at *P* < .05. SPSS software Version 28.0.1.1 (IBM-SPSS) and Jamovi Version 2.3.26 were used for statistical analyses.

## Results

Anteromedial rotatory knee laxity was assessed in 30 knees of 15 healthy individuals (53% male) with a mean age of 26.2 ± 3.5 years (range, 17-31 years) and a mean BMI of 22.5 ± 3.3 (range, 17.7-31.3).

AMT in a healthy population ranged between 2.2 ± 0.8 mm and 3.0 ± 0.9 mm in neutral tibial rotation (Table 1). For each rater, the highest AMT was found in neutral rotation, followed by external tibial rotation and internal tibial rotation. A significant difference in AMT was found between rotational positions of the tibia for all 3 raters.

**Table 1:** Anteromedial Knee Translation^*a*^

	Rater 1	Rater 2	Rater 3
NR, mm	3.0 ± 0.9	2.2 ± 0.8	2.4 ± 0.7
ER, mm	2.4 ± 0.7	2.0 ± 0.6	2.2 ± 0.6
IR, mm	2.2 ± 0.8	1.8 ± 0.6	1.8 ± 0.6
NR vs ER	0.5 (0.15 to 0.92)^*b*^	0.2 (–0.10 to 0.51)	0.2 (–0.10 to 0.57)
NR vs IR	0.8 (0.33 to 1.25)^*c*^	0.4 (0.14 to 0.76)^*b*^	0.6 (0.37 to 0.87)^*c*^
ER vs IR	0.3 (–0.15 to 0.66)	0.2 (–0.10 to 0.56)	0.4 (0.15 to 0.62)^*b*^

a Continuous variables are expressed as mean ± SD. Group comparisons are expressed as mean (95% CI) for between-group differences. Group comparisons were based on a 1-way repeated-measures analysis of variance with Bonferroni-adjusted post hoc analysis. ER, external tibial rotation; IR, internal tibial rotation; NR, neutral tibial rotation.

b Statistically significant difference (*P* < .05).

c Statistically significant difference (*P* < .001).

For AMT measurements, poor to moderate and good to excellent interrater and intrarater reliabilities, respectively, were demonstrated (Table 2). Interrater and intrarater reliabilities for SSD measurements were poor to moderate and moderate to excellent, respectively (Table 3). For AMT and SSD measurements, interrater and intrarater reliabilities were lowest in external tibial rotation.

**Table 2:** Intraclass Correlation Coefficients (ICCs) for Anteromedial Knee Translation

	Interrater ICC (95% CI)	Intrarater ICC (95% CI)		
		Rater 1	Rater 2	Rater 3
Neutral rotation	0.63 (0.31-0.81)	0.94 (0.89-0.97)	0.96 (0.93-0.98)	0.95 (0.91-0.98)
External rotation	0.44 (0-0.71)	0.89 (0.80-0.94)	0.88 (0.79-0.94)	0.92 (0.85-0.96)
Internal rotation	0.73 (0.50-0.86)	0.93 (0.88-0.97)	0.94 (0.88-0.97)	0.92 (0.84-0.96)

**Table 3:** Intraclass Correlation Coefficients (ICCs) for Side-to-Side Differences

	Interrater ICC (95% CI)	Intrarater ICC (95% CI)		
		Rater 1	Rater 2	Rater 3
Neutral rotation	0.56 (0.02-0.84)	0.91 (0.74-0.97)	0.88 (0.66-0.96)	0.96 (0.86-0.99)
External rotation	0.12 (0-0.69)	0.86 (0.61-0.95)	0.61 (0-0.87)	0.63 (0-0.88)
Internal rotation	0.69 (0.30-0.88)	0.87 (0.60-0.96)	0.89 (0.68-0.96)	0.88 (0.64-0.96)

**Figure 2.**
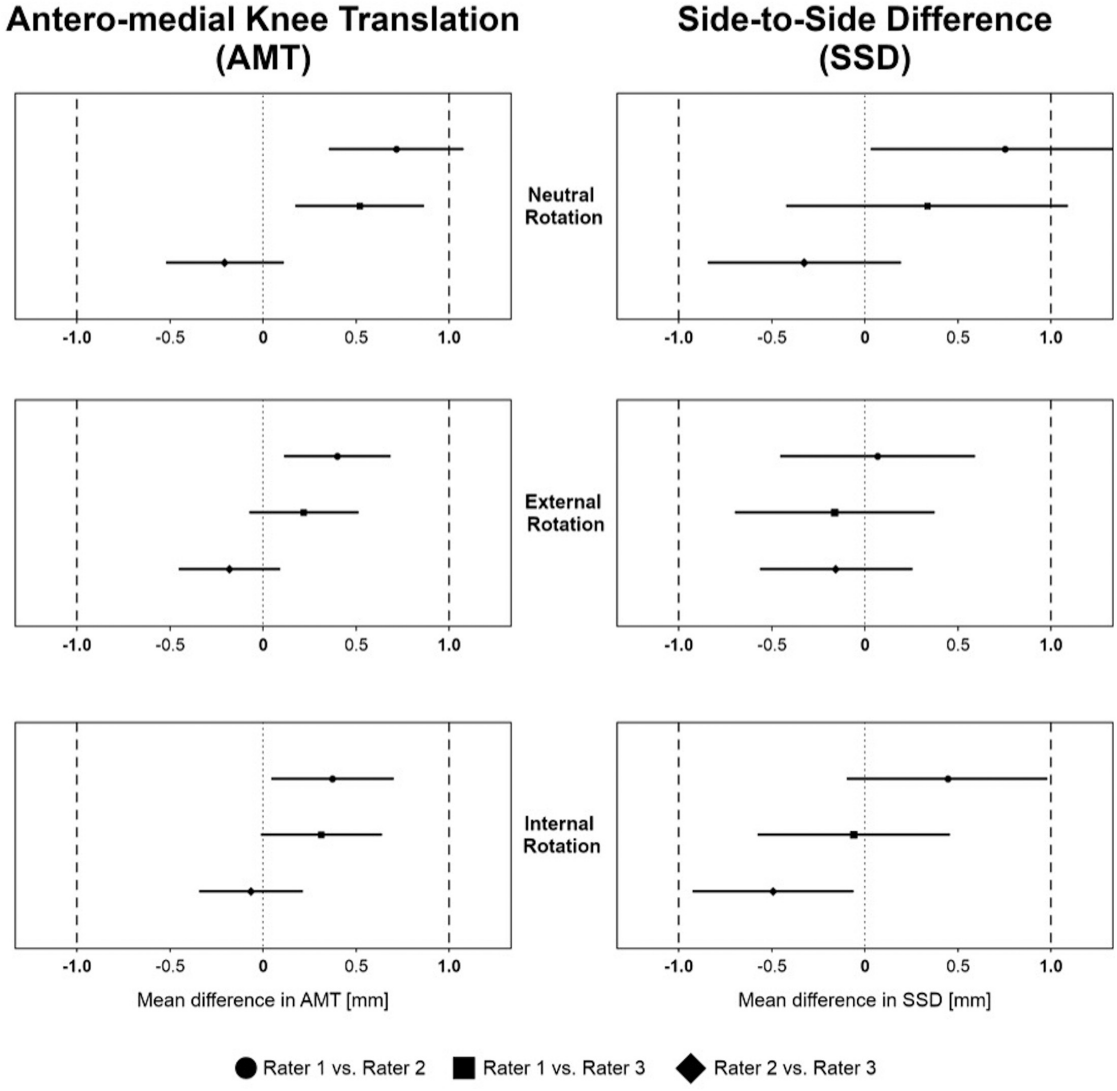
Equivalence for anteromedial knee translation (AMT) and side-to-side difference (SSD) measurements between rater 1 and rater 2, between rater 1 and rater 3, and between rater 2 and rater 3 for neutral, external, and internal tibial rotation. Equivalence boundaries, ±1 mm.

Significant equivalence of AMT and SSD measurements was observed between all raters for external and internal tibial rotation (all *P* < .05) (Figure 2). In neutral tibial rotation, equivalence of AMT measurements was observed between raters 1 and 3 and between raters 2 and 3 (all *P* < .05). Equivalence in SSD measurements in neutral tibial rotation was found between raters 2 and 3 (*P* < .05).

**Figure 3.**
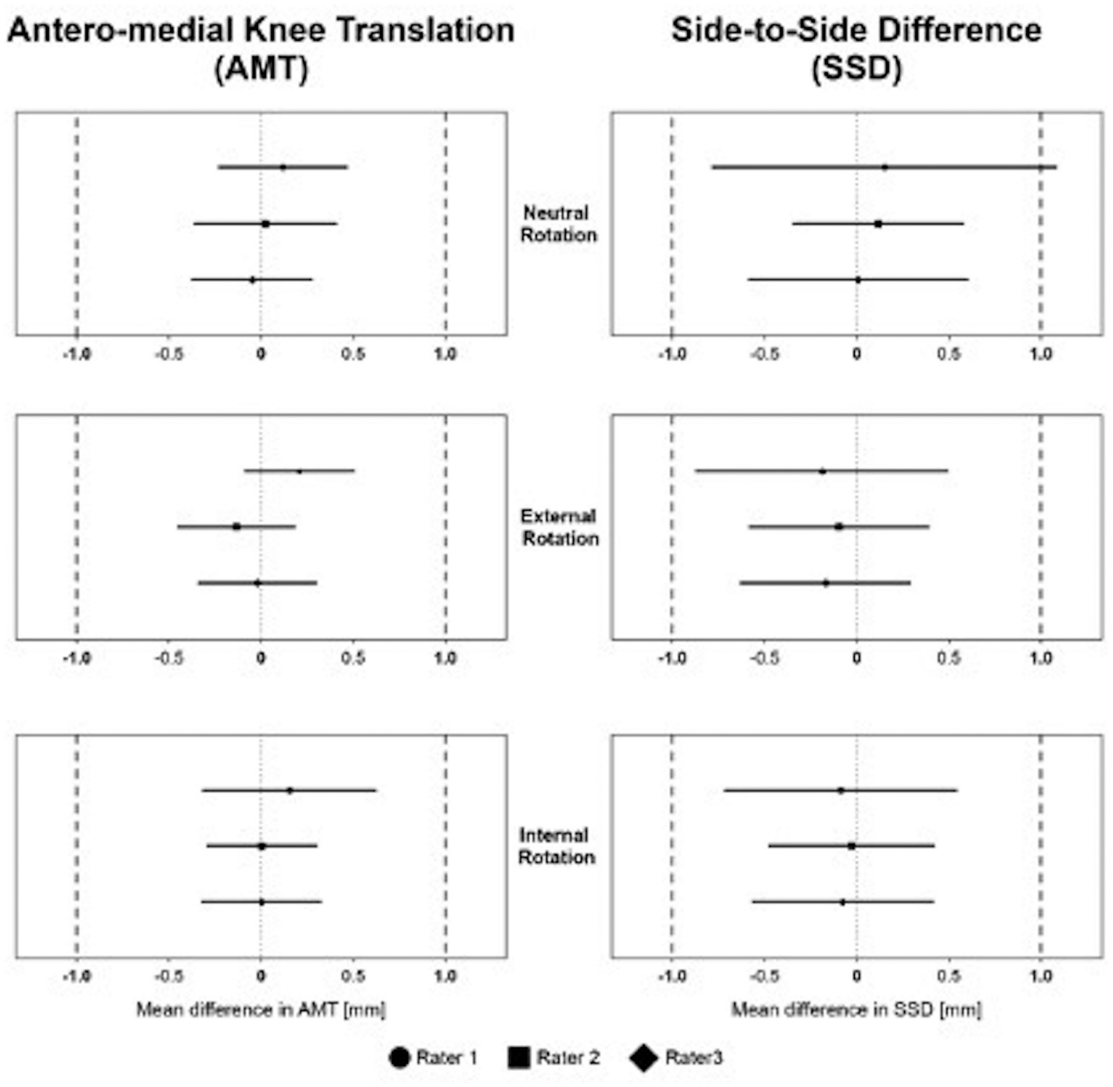
Equivalence for anteromedial knee translation (AMT) and side-to-side difference (SSD) measurements between interval 1 and interval 2 of 1 test cycle of rater 1, rater 2, and rater 3 for neutral, external, and internal tibial rotation. Equivalence boundaries, ±1 mm.

Significant equivalence of AMT and SSD measurements between interval 1 and interval 2 of the corresponding test cycle was observed for each rater in each rotational position of the tibia, except for SSD measurement of rater 1 in neutral tibial rotation (Figure 3).

## Discussion

The most important finding of this study was that anteromedial rotatory knee laxity could be quantified using a noninvasive image analysis software. The study defines standard values that correspond to previous biomechanical measurements.^35,36^ Agreement and equivalence of measurements were good to excellent within raters and poor to moderate between raters. However, measurements were within equivalence boundaries of ±1 mm. Another pivotal finding was that AMT was significantly related to tibial rotation, with the highest AMT observed at neutral tibial rotation.

In recent years, rotatory instability of the knee and concomitant peripheral injuries in addition to ACL tears have gained much research interest. In addition to anterolateral rotatory instability, injuries to the medial knee structures have received increasing attention.^2,31,33,37^ Studies have shown that medial-sided knee instability increases the risk for ACL retears after ACL reconstruction and must be addressed (probably at the initial management) in order to avoid treatment failure.^2,31^ Combined injuries to the ACL and the MCL complex are frequent and occur in 34% to 67% of patients treated with isolated ACL reconstruction.^33,37^ Those injuries can lead to both valgus and anteromedial rotatory instability depending on the injury pattern.^21,22^ The deep MCL and the anterior portion of the superficial MCL are the major restraints for AMRI,^4,9,27^ and injuries of these structures result in a significant increase in anterior laxity of the medial tibial plateau when anterior force or external rotation is applied.^34^ Clinical reports about patients who have AMRI are sparse, as are reports on outcomes after nonsurgical or surgical treatment. This is surprising because several studies have investigated anterolateral rotational instability by clinical examination (eg, pivot-shift test) or by quantifying anterolateral tibial plateau movement using tracking systems.^1,6,13,24,25,28^ With regard to AMRI, Slocum and Larson^30^ described a clinical test performed with the patient in 90° of knee flexion with the foot supported in external rotation, and they graded their findings with regard to the forward tibial displacement. Forward dislocation up to one-half of an inch was graded 1, one-half to three-fourths was graded 2, and more than three-fourths was graded 3.^30^ Wierer et al^34^ evaluated AMRI in a biomechanical study and described a quantification model based on forward displacement of the tibia. The authors classified a displacement of 1 to 5 mm as grade I, 6 to 10 mm as grade II, and >10 mm as grade III. However, objective quantified values for uninjured knees or measurements in case of injury do not exist and clinical results rely only on subjective ratings.

In the present study, AMT was measured in different rotational positions of the tibia to investigate the behavior of the knee in various ligament tensioning states. The application is not able to measure rotation, and therefore AMT and not anteromedial rotatory laxity was the primary outcome. Overall, AMT in a healthy knee was between 1.8 and 3.0 mm depending on both the rotational position and the investigator. The AMT values are in close agreement with biomechanical studies that found anterior tibial translation in cadaveric knees to range between 2 and 3 mm in 90° of knee flexion when an 88-N anterior drawer force was applied.^35,36^ This finding suggests that the PIVOT iPad application measures valid AMT values that correspond to biomechanical findings in a highly standardized environment and can consequently be deemed reliable measurements.

Differences in AMT between rotational tibial positions were small, ranging between 0.4 and 0.8 mm. For each rater, AMT was highest with the tibia in neutral rotation. This finding seems reasonable given that the peripheral knee structures are under less tension compared with internal and external tibial rotation and allow the tibia to move forward.^16,38^ In external tibial rotation, the intact anteromedial structures, including the anteromedial capsule and the anterior fibers of the superficial and deep MCL, become tight and resist AMT.^4,16,38^ In tibial internal rotation, the anterolateral capsule and the iliotibial band become tightened, and the cruciate ligaments wind around each other to resist forward displacement of the tibia.^14,15^ In contrast, a previous study found that tibial anterior translation was highest in tibial external rotation.^8^ The authors observed an anterior displacement between 6.3 mm (internally rotated) and 10.1 mm (externally rotated) by applying an 89-N anterior drawer force in 20° of flexion using a KT-1000 arthrometer. However, measurements were made in patients with ACL injury, and the authors did not report on potential concomitant lesions to the periphery (eg, MCL) or menisci.^8^ It is important to note that joint laxity in 20° of flexion is significantly higher than in 90° of flexion, even in healthy knees.^36^ Mayr et al^20^ showed a higher SSD in tibial external rotation compared with neutral and internal rotation in patients with combined ACL/MCL injuries using a novel device (Laxitester). Runer et al^28^ tested 4 different devices for measuring anterior tibial translation in healthy knees and described an anterior knee laxity of between 5 and 6.5 mm in 25° of flexion when using the Rolimeter. The KiRa system was the only device that showed significantly higher anterior tibial translation values than the other devices tested (Rolimeter, KT-1000, and KLT). In agreement with our results, Runer et al found only poor to moderate intra- and interrater reliability for all devices when testing anterior knee laxity. Nevertheless, and more critically, the standard error of measurement between raters was found to lie within the predefined boundaries of ±1 mm and, hence, was considered accurate enough for measuring AMT in healthy individuals in our study. Even though interrater reliability was low, the equivalence and agreement within raters and between raters were excellent. The differences in the study participants’ native knees were clinically insignificant, as differences <1 mm can hardly be differentiated by clinical examination. This is promising, but future studies are needed that include patients with AMRI to determine how the tibial movement increases with different MCL injury patterns and whether these injuries can be quantified using the PIVOT iPad application.

The PIVOT iPad application has been shown to reliably facilitate noninvasive measurements of rotational knee laxity and has been used in many previous studies.^10,12,13,24,25^ It eliminates the need for radiation or specialized equipment, is easily accessible and cost-effective, and provides objective measurements of instability and pre- and postoperative laxity. To date, it has only been used to assess anterolateral rotatory knee laxity during pivot-shift maneuvers and has not been used for other rotational laxities. The present study shows that the application can also reliably quantify AMT on the medial side during the Slocum test in healthy individuals.

The PIVOT application may be a promising tool for quantifying anteromedial rotatory instability of the knee. The quantification of AMRI using the PIVOT iPad application opens new avenues for research and clinical management. Longitudinal studies can be conducted to investigate the natural history of AMRI and its correlation with functional outcomes. The PIVOT's accessibility, real-time measurement capabilities, and potential for remote monitoring make it a valuable addition to the clinician's toolkit.

This study must be seen in conjunction with its limitations. To the best of our knowledge, this is the first study to investigate AMT using an image analysis software, which has been validated only for anterolateral rotatory instability and the pivot-shift test. Therefore, it was not possible to compare the results with previous studies measuring AMRI. A primary limitation of this study was that patients with ACL and MCL injuries were not included in the study. The study provides only baseline control data related to laxity measures and does not provide insight into how the PIVOT app performs to measure instability. Another limitation is that the number of participants was small, which may introduce the potential for a type II error. However, the standard deviation was small, and an a priori sample size calculation showed that the study had enough power. The tibial adhesive markers were fixed not to the skin but rather to a circumferential strap, which entails the risk of movements between the strap and skin in addition to motion between skin and anatomic landmarks. No imaging data were obtained, which could have shown nonsymptomatic injuries that could influence the assessment of AMRI.

This study provides a basis for future clinical research on anteromedial rotatory knee laxity by quantifying tibial forward displacement in healthy individuals and defining standard values.

## Conclusion

Anteromedial rotatory knee laxity in uninjured individuals was quantified using a noninvasive image analysis software, and the highest AMT was observed during neutral tibial rotation. Reliability and equivalence of measurements were good to excellent within raters and poor to moderate between raters. Therefore, repeated AMT measurements are recommended to be performed by the same investigator. This simple, cost-effective, and readily available tool may support surgeons in surgical decision-making regarding anteromedial knee instability.
